# New Insights into the Specificity, Authenticity, and Traceability Analysis of Olive Oils

**DOI:** 10.3390/foods10102372

**Published:** 2021-10-06

**Authors:** Raquel Garcia, Maria João Cabrita

**Affiliations:** MED—Mediterranean Institute for Agriculture, Environment and Development, Departamento de Fitotecnia, Escola de Ciências e Tecnologia, Universidade de Évora, Pólo da Mitra, Ap. 94, 7006-554 Évora, Portugal; raquelg@uevora.pt

Olive oil is a traditional product of the Mediterranean diet. The nutritional properties and remarkable health benefits associated with olive oil consumption explain the growing worldwide use of this product in people’s diets [1]. In fact, olive oil consumption has spread to countries that were not traditionally olive oils producers. Owing to its high price, extra virgin olive oil—the top grade of olive oil production—could be prone to mislabeling and adulteration practices. This constitutes economic fraud but generally does not represent a human health risk. However, this is a global issue that damages the reputation of companies, disrupts markets, and leads to a loss of consumer confidence. Despite the protection of this edible oil by several EU regulations (e.g., regulation (EU) No 1308/2013, regulation (EEG) No 2568/91, or regulation (EU) No 29/2012), olive oil is still one of the top ten products most affected by adulteration practices. These regulations enable us to standardize the classification of different olive oil categories, but authenticity and traceability cannot be addressed by conventional methods. Nowadays, the specificity, authenticity, and traceability of olive oils are prominent topics. Aware of this concern, the scientific community is exploring the development and implementation of analytical tools and methodologies that are suitable for accomplishing these tasks. Indeed, several innovative approaches are emerging, allowing to implement more selective, reliable, and robust methods. Thus, this Special Issue aims to highlight emerging topics related to the specificity, authenticity, and traceability analysis of olive oils and allow researchers to present their latest developments and innovations to a global audience.

Despite the aim of this Special Issue encompassing three key topics—specificity, authenticity, and adulteration—no contributions were received regarding the theme of specificity. Thus, this editorial has collected papers giving an interesting outlook on several promising methodologies, including NMR-based approaches [2,3], chromatographic techniques [4,5,6,7], and stable isotope analysis [8] combined with advanced statistical modelling techniques for addressing the geographic and varietal origin of olive oils and cultivar differentiation, highlighting the themes of authenticity and traceability. Researchers have also devoted their attention to some classes of target compounds that are present in olive oils, namely volatiles [6], n-alkanes and n-alkenes [5], triacylglycerols [2], and biophenols [7]. Their role in the discrimination between olive oils based on geographic origin and cultivar, using advanced analytical techniques, has been investigated. Another approach encompassed in this Special Issue covers the building of a specific ^1^HNMR profiling database using monocultivar reference olive oil for the analysis of olive oil blends [3]. Additionally, to verify the geographic origin of virgin olive oil, an untargeted analysis using LC-qToF-MS was utilized in one study [4].

All these papers actively contribute to creating a more complete picture of the situation and could open new avenues in the field of food authenticity and traceability.
